# Oral ondansetron for paediatric gastroenteritis in primary care: a randomised controlled trial

**DOI:** 10.3399/BJGP.2021.0211

**Published:** 2021-08-24

**Authors:** Irma J Bonvanie, Anouk AH Weghorst, Gea A Holtman, Heleen A Russchen, Freek Fickweiler, Henkjan J Verkade, Boudewijn J Kollen, Marjolein Y Berger

**Affiliations:** Department of General Practice and Elderly Care Medicine, University of Groningen, The Netherlands.; Department of General Practice and Elderly Care Medicine, University of Groningen, The Netherlands.; Department of General Practice and Elderly Care Medicine, University of Groningen, The Netherlands.; Department of General Practice and Elderly Care Medicine, University of Groningen, The Netherlands.; Department of General Practice and Elderly Care Medicine, University of Groningen, The Netherlands.; Department of Pediatrics, University of Groningen, The Netherlands.; Department of General Practice and Elderly Care Medicine, University of Groningen, The Netherlands.; Department of General Practice and Elderly Care Medicine, University of Groningen, The Netherlands.

**Keywords:** acute gastroenteritis, child, oral ondansetron, out of hours, primary care, vomiting

## Abstract

**Background:**

Acute gastroenteritis (AGE) affects almost all children aged ≤5 years. In secondary care, ondansetron was found to be effective at reducing vomiting.

**Aim:**

To determine the effectiveness of adding oral ondansetron to care as usual (CAU) to treat vomiting in children with AGE attending out-ofhours primary care (OOH-PC).

**Design and setting:**

A pragmatic randomised controlled trial at three OOH-PC centres in the north of the Netherlands (Groningen, Zwolle, and Assen), with a follow-up of 7 days.

**Method:**

Children were included if they were: aged 6 months–6 years; AGE diagnosed by a GP; ≥4 reported episodes of vomiting in the 24 hours before presentation; ≥1 reported episode of vomiting in the 4 hours before presentation; and written informed consent from both parents. Children were randomly allocated to either the control group or the intervention group. The control group received CAU, namely oral rehydration therapy. The intervention group received CAU plus one dose of oral ondansetron (0.1 mg/kg).

**Results:**

In total, 194 children were included for randomisation. One dose of oral ondansetron decreased the proportion of children who continued vomiting within 4 hours from 42.9% to 19.5%, with an odds ratio of 0.37 (95% confidence interval [CI] = 0.20 to 0.72, number needed to treat: four). Ondansetron also decreased the number of vomiting episodes within 4 hours (incidence rate ratio 0.51 [95% CI = 0.29 to 0.88]) and improved overall parental satisfaction with treatment (*P* = 0.027).

**Conclusion:**

Children with AGE and increased risk of dehydration due to vomiting could be treated with ondansetron in primary care to stop vomiting more quickly and increase parental satisfaction with treatment. These results could be used to improve the quality and efficacy of general practice medicine.

## INTRODUCTION

Acute gastroenteritis (AGE) is common in young children and, although it is typically self-limiting, severe dehydration is an important complication.^1^ Approximately 5% of all GP consultations with children in the Netherlands are for AGE.^2^ Among those seen in primary care, 8.1% are referred to specialist care and 8000 are admitted to the hospital each year.^2^^,^^3^ However, it is thought that many of these referrals and admissions can be avoided.^4^

International guidelines recommend care as usual (CAU) with oral rehydration therapy (ORT) to prevent and treat dehydration in children.^5^ It has been shown that prescribing ORT with education can reduce hospital admission by up to 45%,^4^^,^^6^^–^^8^ yet it is still underused in primary care; indeed, only 4% of all children overall with AGE received ORT through their GP.^9^^,^^10^ A suggested reason for this underuse is that 70% of these children present with vomiting as the predominant symptom.^9^ National paediatrics guidelines mention persistent vomiting as a predictor of ORT failure in children who are dehydrated;^11^ as such, most GPs are less likely to prescribe ORT when the child predominantly presents with vomiting.^12^

Ondansetron has been reported to be safe and effective at stopping vomiting, increasing ORT success, and reducing hospitalisation rates among children presenting with AGE in secondary care;^13^ however, the practical value of ondansetron for treating children with AGE in primary care is unknown. The authors aimed to conduct a pragmatic randomised controlled trial (RCT) to investigate the effect of ondansetron: added to CAU; compared with CAU alone; and on vomiting in children aged 6 months–6 years with AGE consulting out-of-hours primary care (OOH-PC) services.

## METHOD

### Study design

Participants were enrolled from December 2015 until January 2018 at three OOH-PC centres in the north of the Netherlands: one in Groningen, one in Zwolle, and one in Assen. A detailed description of the study design, recruitment strategy, outcomes, and discussion of the informed consent procedure are described elsewhere.^14^ This study started with a pilot (Dutch Trial Register reference number: NL4700) undertaken from December 2015 until October 2016 but, as a result of the low inclusion rate, the primary outcome was changed from ‘referrals’ to ‘vomiting’. In agreement with the Medical Ethics Review Committee of the University Medical Center Groningen, children included from the pilot were also included in the new trial and the RCT was approved.

### Inclusion and exclusion criteria

Children considered to be at increased risk of dehydration^15^ were included if they met the following inclusion criteria:

- aged 6 months–6 years;
- diagnosis of AGE confirmed by a GP at the OOH-PC centre;
- ≥4 reported episodes of vomiting 24 hours prior to presentation; and
- ≥1 reported episode of vomiting 4 hours prior to presentation.

**Table:** How this fits in

Ondansetron was found to be effective at reducing vomiting in children with AGE in secondary care, but this effect has never been evaluated in primary care. Based on the findings of this study, ondansetron use is effective at dramatically reducing vomiting, seems safe, and is positively evaluated by parents when used to treat children aged ≤5 years with acute gastroenterisits (AGE). As such, ondansetron could be considered by GPs as an additional treatment in the management of dehydration due to AGE, when the child is predominantly vomiting. Future research should disentangle the key factors leading to hospital referrals and consider ways to administer oral rehydration therapy more effectively in primary care or at home.

Children who met the following criteria were excluded:

- used, or prescribed, antiemetics in the previous 6 hours;
- known renal failure or hypoalbuminemia;
- known diabetes mellitus or inflammatory bowel disease;
- history of abdominal surgery that could explain the current symptoms (according to the GP);
- known sensitivity to 5-HT3 receptor antagonists;
- known prolonged QT interval, or current use of QT-prolonging medication; and
- previous enrolment in the study.

Additionally excluded were those children for whom no extended written informed consent of the second parent was received. Exclusion on this basis was performed after randomisation because of protocol violation as set by the university’s Medical Ethics Review Committee.

### Randomisation and blinding

Randomisation occurred after written informed consent was obtained from the consulting parent plus verbal informed consent from the second parent (in most cases they were at home).

After consent was gained, children were randomly allocated to one of two intervention groups at a ratio of 1:1. An online randomisation tool was used to generate the allocation sequence in direct response to participant inclusion by the research assistant; concealment was not an issue because allocation was only generated after randomisation. The allocation sequence was stratified by age (6–24 months or >24 months) and severity of dehydration (‘at risk’ for no alarm symptom or ‘dehydrated’ for ≥1 alarm symptom). Comparisons between groups were adjusted for these stratification factors.

Participants, parents, GPs, and research assistants were not blinded to the allocated treatment. Ondansetron has already been proven effective at reducing vomiting in blinded RCTs.^16^^,^^17^ In this pragmatic RCT, the authors specifically aimed to investigate the potential effect of implementing ondansetron in routine primary care; blinding participants would, in this case, result in outcomes not translatable to daily practice. The statistician performing the analyses was blinded to the treatment allocation by an independent researcher. The primary outcome was not known by parents and GPs.

### Interventions

#### Control group: CAU

CAU comprised instructions to buy oral rehydration solution and how to use it, as described in the acute diarrhoea guideline of the Dutch College of General Practitioners:^15^ 10 ml/kg compensation when at risk of dehydration (that is, all children) and 15 ml/kg for 4 hours if a GP assessed the patient as being dehydrated. The research assistant provided the parents the instructions with a patient folder containing the same information, discussed alarm symptoms, and advised them to contact the GP if there was no improvement or symptoms worsened.^15^

#### The intervention: CAU plus ondansetron

Children allocated to the intervention group received the CAU described above plus a single weight-based dose of oral ondansetron syrup (0.1 mg/kg), in accordance with the Dutch Pediatrics Formulary.^18^ If the child vomited within 15 min of administration, the same dose was repeated once, but a third dose was not given.

### Outcomes

Parents completed diaries for 7 days. For the first 4 hours after presentation, they reported hourly; thereafter, they reported daily until 7 days after presentation. If parents did not return the diary after multiple requests, information about the primary outcome was collected by telephone.

#### Primary outcome

The primary outcome was the proportion of children who continued vomiting in the first 4 hours after randomisation. This evaluation point was chosen because the circulating concentration of ondansetron is expected to reach 50% of its maximum serum level at 3 hours after oral dosing, meaning that direct effects on vomiting are unlikely beyond 4 hours.^18^ In addition, national guidelines recommend that GPs evaluate the effect of treatment on symptoms and assess the indications for referral in children with AGE by 4 hours after initial presentation.^8^^,^^11^^,^^15^

#### Secondary outcomes

The following outcomes were assessed up to 4 hours after randomisation:

- number of vomiting episodes per child;
- ORT intake (ml) per participant; and
- proportion of children who experienced ≥1 adverse event(s) related to ondansetron.

The following outcomes were assessed up to 7 days after randomisation:

- proportion of children referred to specialist care; and
- proportion of children admitted to hospital.

Finally, parental satisfaction with ondansetron therapy was assessed using a five-point Likert scale.

### Statistical methods

#### Sample size

Based on a systematic review,^13^ it was estimated that 85% of children in the CAU group and 64% of children in the intervention group would continue vomiting within 4 hours. It was calculated that 100 children per group were needed to achieve an alpha of 0.05 and a power of 0.90. To compensate for an expected loss to follow-up of 10%, the authors aimed to include 220 children.^19^^,^^20^ For the intention-to-treat (ITT) analysis, the authors were able to include 88 and 87 children in the intervention and control groups, respectively; therewith, the power remained >80% (sample size *n* = 166).

#### Handling of missing data

Using logistic regression, the authors explored whether baseline characteristics were related to missing values relating to their outcomes. For all single outcomes, further inspection of frequencies and distribution of values gave no indication that the missing values were related to the true values themselves (that is, values were distributed as theoretically expected). In addition, Little’s^21^ Missing Completely at Random test was not statistically significant (*P*-value χ^2^ 0.76); thus, it was assumed that the missing data were missing at random. Supplementary Table S1 gives an overview of the baseline characteristics of complete cases versus participants with missing values.

All available participant data were entered as predictors in multiple imputation: baseline characteristics, outcomes, and any available variables potentially related to outcomes. After analyses on 20 separate multiple imputed datasets, the results were pooled. In line with the Strengthening the Reporting of Observational Studies in Epidemiology (STROBE) and Consolidated Standards of Reporting Trials (CONSORT) guidelines, all analyses were also performed on cases with complete data only.

#### Main analyses

Data were analysed on both an ITT and a per protocol (PP) basis. In addition, analyses were performed on both multiple imputed data and complete cases. It was assumed that the pooled estimates of ITT analyses on the multiple imputed data would be most reliable and, as such, these were considered the main analyses. All analyses were performed using IBM SPSS Statistics (version 25).

The ITT population consisted of all patients randomly allocated to one of the two treatment groups, regardless of whether they received, or adhered to, the allocated intervention. The only excluded participants were those who did not meet the inclusion criteria or met the exclusion criteria (that is, no informed consent of the second parent or retraction of informed consent).

The PP population consisted of the ITT population, but also excluded participants if they did not receive treatment, deviated from the protocol, or withdrew from the study.

#### Primary and secondary outcome analyses

In all analyses, the treatment (intervention) group was the independent predictor. The primary outcome (continued vomiting) was evaluated by logistic regression, and because all included participants vomited at baseline, analyses were not adjusted for baseline status. The secondary outcome of the number of vomiting episodes was analysed with a log-linear negative binomial model. The secondary outcomes of summed millilitres of ORT intake and parental satisfaction were analysed with a Mann–Whitney *U* test. Other secondary outcomes — ‘referred’, ‘admitted’, and ‘adverse events’ — were evaluated with logistic regression.

#### Sensitivity analyses

Sensitivity analyses were performed on the pre-specified primary and secondary outcome of number of vomiting episodes, excluding the first hour (that is, from 2–4 hours only).

## RESULTS

### Study participants

**Figure 1.**
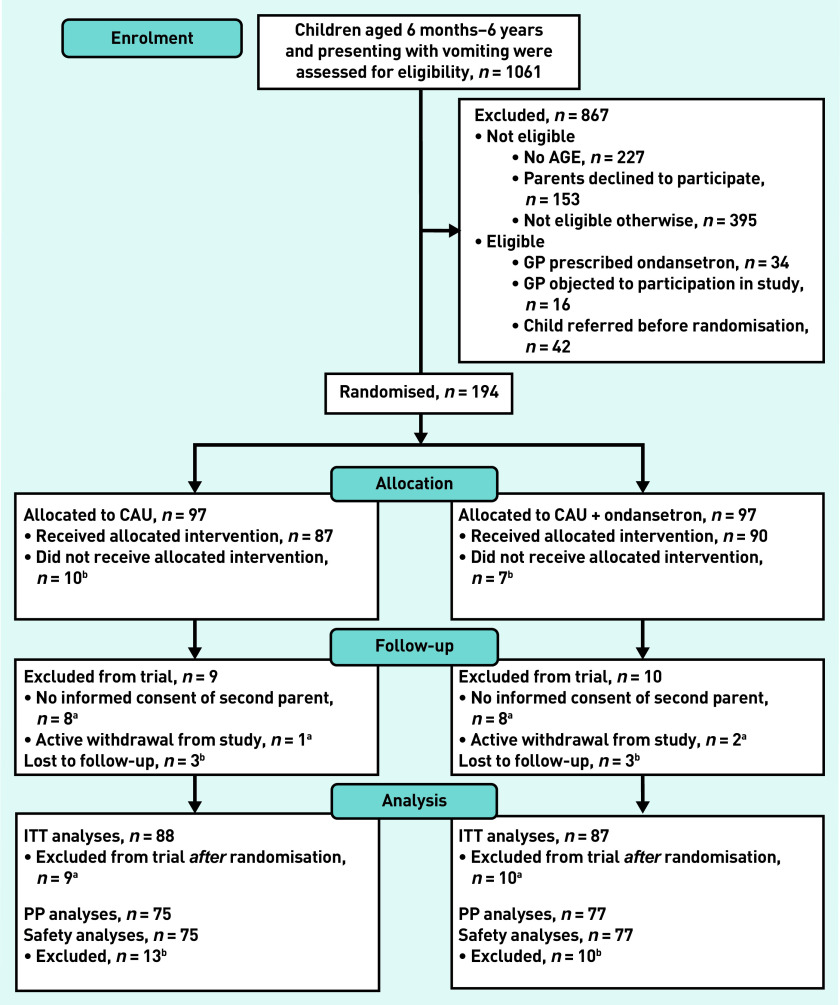
***Participant pathway.*** *^a^* ***Excluded from trial because of no informed consent of second parent (n = 8) or active withdrawal from study (retracted informed consent) (n = 1).*** *^b^* ***Excluded from PP and safety analyses because participants did not receive the allocated intervention (n = 10) or data were lost to follow-up (n = 3). AGE = acute gastroenteritis. CAU = care as usual. ITT = intention to treat. PP = per protocol.***

In total, 1061 participants aged 6 months–6 years who presented with vomiting at one of the three participating primary care OOH-PC centres were screened. Of these, 867 children were excluded: no diagnosis of AGE (*n* = 227) and not eligible because the intention was to include children at increased risk of dehydration (*n* = 395) were the most common reasons for exclusion. Of the remainder, 194 children were included and randomised, 97 of these each formed the CAU and intervention groups (Figure 1).

Sixteen cases were excluded because parents did not return their written informed consent forms, despite initially giving their oral informed consent, and three parents withdrew informed consent after randomisation. As such, data for 175 participants were available for ITT analysis. Seventeen children did not receive the allocated intervention and six were lost to follow-up, resulting in 152 participants available for the PP analyses (Figure 1).

Included participants had a median age of 1.5 years (range: 6 months–6 years), 50.3% were female, the median duration of vomiting before presentation was 2 days (range 0.8–9.0 days), and 71.3% had diarrhoea. There were no statistical differences in baseline characteristics between the CAU and the intervention groups in either the ITT (Table 1) or the PP (Supplementary Table S2) populations.

**Table 1.** Baseline characteristics of the intention-to-treat population

**Baseline characteristic**	**Valid, *n***	**All participants, *n*= 175**	**Valid, *n***	**CAU, *n* = 88**	**Valid, *n***	**Intervention, *n*= 87**
**Age, years, median (IQR)**	175	1.5 (0.9–2.1)	88	1.5 (0.9–2.0)	87	1.5 (0.9–2.2)

**Female, *n* (%)**	175	88 (50.3)	88	50 (56.8)	87	38 (43.7)

**Weight, kg, median (IQR)**	169	11.0 (9.5–14.0)	86	11.0 (9.4–14.0)	83	12.0 (9.5–14.3)

**Duration of vomiting prior to presentation, days, median (IQR)**	174	2.0 (1.0–3.0)	87	1.2 (1.0–2.0)	87	2.0 (1.0–3.0)

**Frequency of vomiting in past 24 hours, median (IQR)**	171	5.0 (4.0–10.0)	86	5.0 (4.0–10.0)	85	6.0 (4.0–10.0)

**Diarrhoea present, *n* (%)**	174	124 (71.3)	87	66 (75.9)	87	58 (66.7)

**Duration of diarrhoea prior to presentation, days, median (IQR)^a^**	124	2.0 (1.0–3.0)	66	1.0 (0.4–2.0)	58	1.0 (0.0–3.0)

**Frequency of diarrhoea in past 24 hours, median (IQR)^a^**	123	3.0 (2.0–5.0)	66	2.0 (1.0–5.0)	57	1.5 (0.0–4.0)

**Dehydration assessed at 0–100% by GP, median (IQR)**	170	20.0 (10.0–40.0)	85	20.0 (6.0–40.0)	85	20.0 (10.0–40.0)

**Use of concomitant medication, *n* (%)**	175	65 (37.1)	88	31 (35.2)	87	34 (39.1)

**Additional risk factors of dehydration, *n* (%)^b^**						
1	175	63 (36.0)	88	33 (37.5)	87	30 (34.5)
≥2	175	18 (10.3)	88	10 (11.4)	87	8 (9.2)

**Alarm symptoms of severe dehydration, *n* (%)^c^**						
1	175	32 (18.3)	88	15 (17.0)	87	17 (19.5)
≥2	175	2 (1.1)	88	1 (1.1)	87	1 (1.1)

a *Numbers only presented for those participants with diarrhoea.*

b *Risk factors assessed at baseline were:* ≥*6 watery stools or diarrhoea, fever, and reduced intake of liquid/food.*

c *Alarm symptoms assessed at baseline were: confused or decreased consciousness, bradycardia, weak peripheral heartbeat pulsations, capillary refill time* >*4 seconds, skin pinch test* >*4 seconds, cold or marbled extremities, and no urine output in the previous 24 hours. CAU = care as usual; IQR = interquartile range.*

The most common risk factor was fever (24.9%) and the most common alarm symptom was no urine output for 24 hours (14.3%) (data not shown).

There was a wide range of missing data for the variables used in the composite measures (12%–49%); in total, 154 participants (88.0% of the 175 included children) provided all data needed for the primary outcome measure (Table 2).

**Table 2.** The effect of ondansetron on primary and secondary outcomes of the intention to treat population

**Effect of ondansetron on primary and secondary outcomes**	**Valid, *n***	**All, participants, *n*= 175**	**Valid, *n***	**CAU, *n*= 88**	**Valid, *n***	**Intervention, *n*= 87**	**Valid, *n***	**Imputed cases, OR (95% CI)**	**Non-imputed cases, OR (95% CI)**
Continued vomiting, hours 1-4, *n* (%)	154	48 (31.2)	77	33 (42.9)	77	15 (19.5)	154	**0.37 (0.20 to 0.72)**	**0.32 (0.16 to 0.66)**
Vomiting episodes, hours 1–4, median (range)^a^	137	0.0 (0.0–6.0)	67	0.0 (0.0–6.0)	70	0.0 (0.0–5.0)	137	**IRR 0.51 (0.29 to 0.88)**	IRR 0.46 (0.21 to 1.03)
Intake ORT, mL, median (IQR)	88	10.0 (0.0–100.0)	46	0.0 (0.0–72.0)	42	35.0 (0.0–180.0)	88	*P* = 0.522^b^	*P* = 0.093^b^
Referrals, *n* (%)	144	28 (19.4)	73	14 (19.2)	71	14 (19.7)	144	1.19 (0.60 to 2.36)	1.04 (0.45 to 2.36)
Hospital admissions, *n* (%)	132	19 (14.4)	73	10 (13.7)	59	9 (15.3)	132	1.80 (0.91 to 3.55)	1.13 (0.43 to 3.00)
Adverse events, *n* (%)^c^	96	30 (31.3)	48	19 (39.6)	48	11 (22.9)	96	0.63 (0.34 to 1.17)	0.45 (0.19 to 1.10)
Serious adverse events, *n* (%)^d^	91	6 (6.6)	46	4 (8.7)	45	2 (4.4)	91	0.83 (0.45 to 1.54)	0.49 (0.09 to 2.81)
Parental satisfaction, median (IQR)	107	4.0 (3.0–4.0)	53	4.0 (3.0–4.0)	54	4.0 (4.0–5.0)	107	***P* = 0.027** ^b^	***P* = 0.013** ^b^

a *Complete range provided instead of IQR because data are heavily skewed (IQR = 0–0).*

b *Mann–Whitney* U *test.*

c *Adverse events: erythema, hiccups, and headache.*

d *Serious adverse events: spasms/convulsions and breathing problems. Bold = statistically significant difference. CAU = care as usual. IQR = interquartile range. IRR = incidence rate ratio. OR = odds ratio. ORT = oral rehydration therapy.*

### Outcomes

#### The effect of ondansetron on continued vomiting and vomiting episodes

The pooled estimates of ITT analyses on the multiple imputed data were considered as the main analyses. Ondansetron decreased the proportion of children who continued vomiting within the first 4 hours after randomisation from 42.9% to 19.5% (Table 2). This corresponded with a relative risk of 0.60 (95% confidence interval [CI] = 0.45 to 0.81) and number needed to treat of four (odds ratio [OR] 0.37, 95% CI = 0.20 to 0.72). In the intervention group, children had fewer vomiting episodes within the 4 hours after randomisation when compared with the CAU group; the incidence rate ratio (IRR) was 0.51 (95% CI = 0.29 to 0.88) (Table 2). Similar estimates were found when repeating the analysis in the PP population (Supplementary Table S3).

#### The effect of ondansetron on ORT intake, referrals, and hospital admissions

Intake of ORT, number of referrals, and number of hospital admissions did not statistically significantly differ between treatment groups. In both treatment groups, the median ORT intake within 4 hours was 10 ml, referral occurred for 19.4% of all children, and most referred children (74.0%) were admitted to hospital (data not shown). Of all included children, 14.4% were admitted to hospital (Table 2).

#### Associated adverse events and parental satisfaction with ondansetron

Ondansetron did not increase the occurrence of adverse events. The median parental satisfaction with treatment after 1 week was statistically significantly higher in the intervention group 4.0 (interquartile range [IQR] 4.0–5.0) than in the CAU group 4.0 (IQR 3.0–4.0), respectively (*P* = 0.027) (Table 2).

### Sensitivity analyses

In the sensitivity analysis, the effect of ondansetron on continued vomiting during the first 4 hours after randomisation remained statistically significant (OR 0.44, 95% CI = 0.23 to 0.87), but the number of vomiting episodes did not differ between treatment groups (IRR 0.62, 95% CI = 0.34 to 1.13) (data not shown).

## DISCUSSION

### Summary

One dose of ondansetron given in an OOHPC setting decreased the proportion of participants with AGE who had persistent vomiting by 54.5% (decreased from 42.9% [ *n* = 33/88] to 19.5% [ *n* = 15/87] = 54.5% reduction). Overall, ORT intake was low (10 ml/4 hours) and referral rates were high (19% in comparison with a mean referral rate of 8.1%).^3^ Ondansetron use did not appear to increase ORT intake or lead to fewer hospital referrals or admissions; nevertheless, parents were more satisfied with the addition of ondansetron compared with ORT alone.

### Strengths and limitations

The authors are aware of no other studies investigating the practical effectiveness of ondansetron on vomiting and other important treatment goals in children with AGE, when parents consult in an OOH-PC setting. Other strengths of this study are that nearly 600 GPs collaborated over a period of >2 years, and that it was possible to gather data about the reasons for exclusion. From these data, it becomes clear that the intention to select the subgroup of children who, at presentation, frequently vomited was fulfilled. In addition, the use of an hourly diary for the first 4 hours provided detailed and reliable data on the primary outcome.

Limitations of the study were that there was a wide range of missing values measures. Although no association was found between missing values and either treatment, the findings based on these secondary outcome measures should be interpreted with caution. It could also be seen as a limitation that participants — that is, parents and GPs — were not blinded for the intervention. Although it is disputable whether this would have been desirable in a pragmatic trial, the authors believe it did not influence the primary outcome measurement as the aim was to investigate the potential effect of implementing ondansetron in routine primary care and the outcome assessors were blinded.

### Comparison with existing literature

The finding that oral ondansetron reduces the incidence of vomiting and the proportion of vomiting episodes within 4 hours after presentation at an OOH-PC centre is consistent with results of other studies.^13^^,^^22^ The findings presented here also indicate that this effect of ondansetron on vomiting persisted over a 4-hour period.

In addition, the results indicate that a 0.1 mg/kg dose of ondansetron in primary care is at least comparably effective at inducing vomiting cessation as a higher dose given in the emergency department.^13^ Despite ORTs being prescribed for all children included by research assistants, the reported ORT intake was low in both treatment groups for the current study. Studies from emergency department settings indicate that ORT can have a success rate of 100% when prepared and administered by qualified and trained nurses directly after giving a dose of ondansetron.^23^

It would be interesting to study alternatives to ORT that children can better tolerate or accept at home, such as diluted apple juice.^24^ However, for the CAU group, the guideline of the Dutch College of General Practitioners was followed,^15^ which does not include the use of apple juice.

There could be several reasons for the high referral rate among children with AGE and frequent vomiting; a plausible explanation may be that it reflects a lack of success with ORT at home. In the current study, the median intake of oral rehydration solution of 10 ml in 4 hours was considered ineffective for children at any age. Finding ways to improve ORT success at home seems to be key to rectifying this issue. In addition, because vomiting cessation did not lower referral rates, the decision to refer a child with AGE may have been influenced by considerations other than risk factors for dehydration and hydration status.

Such factors may include how parents interpret and communicate symptoms of dehydration, the related healthcare-seeking behaviour of parents, and how exactly GPs follow up on their paediatric patients after discharge from the OOH-PC setting.^25^

Treatment groups had comparable rates of adverse events consistent with the findings of a systematic review and meta-analysis,^17^ which showed that the number and type of adverse events was comparable between oral ondansetron and placebo groups, with no serious adverse events. Although the use of ondansetron in primary care seems safe, further monitoring and reporting for potential side-effects is still indicated when it is prescribed.

### Implications for practice and research

In this study, ondansetron use was found to be effective, safe, and positively evaluated by parents when used to stop vomiting among children aged 6 months–6 years presenting in primary care with AGE and vomiting.

As such, the authors advocate that ondansetron be considered an add-on treatment for use by GPs when managing dehydration due to AGE and frequent vomiting in primary care. However, the findings also show that ondansetron alone will not substantially affect ORT intake or reduce the high referral rate to specialised care.

Future research should aim to disentangle the key factors leading to hospital referral for children with AGE. Research should also consider ways to administer ORT more effectively in primary care or at home, such as direct administration by nurses, better parental education, and the use of alternatives for ORT.

## References

[1] De Wit MA, Koopmans MP, Kortbeek LM (2001). Sensor, a population-based cohort study on gastroenteritis in the Netherlands: incidence and etiology. Am J Epidemiol.

[2] van Pelt W, Friesema I, Doorduyn Y (2009). Trends in gastro-enteritis in Nederland: notitie met betrekking tot 2007[Trends in gastroenteritis in the Netherlands: note related to 2007].

[3] Wolters PI, Holtman G, Fickweiler F (2020). Referral rates for children with acute gastroenteritis: a retrospective cohort study. BJGP Open.

[4] McConnochie KM, Russo MJ, McBride JT (1999). How commonly are children hospitalized for dehydration eligible for care in alternative settings. Arch Pediatr Adolesc Med.

[5] van den Berg J, Berger MY (2011). Guidelines on acute gastroenteritis in children: a critical appraisal of their quality and applicability in primary care. BMC Fam Pract.

[6] Dalby-Payne JR, Elliott EJ (2011). Gastroenteritis in children. BMJ Clin Evid.

[7] Freedman SB, Pasichnyk D, Black KJL (2015). Gastroenteritis therapies in developed countries: systematic review and meta-analysis. PLoS One.

[8] Zolotor AJ, Randolph GD, Johnson JK (2007). Effectiveness of a practice-based, multimodal quality improvement intervention for gastroenteritis within a Medicaid managed care network. Pediatrics.

[9] Chow CM, Leung AKC, Hon KL (2010). Acute gastroenteritis: from guidelines to real life. Clin Exp Gastroenterol.

[10] van der Linden MW, Westert GP, de Bakker D, Schellevis F (2004). Tweede Nationale Studie naar ziekten en verrichtingen in de huisartspraktijk: klachten en aandoeningen in de bevolking en in de huisartspraktijk [Second National Study into diseases and procedures in general practice: complaints and disorders in the population and in general practice].

[11] (2013). Nederlandse Vereniging voor Kindergeneeskunde [Dutch Association for Pediatrics]. NVK richtlijn: dehydratie.

[12] Ozuah PO, Avner JR, Stein REK (2002). Oral rehydration, emergency physicians, and practice parameters: a national survey. Pediatrics.

[13] Carter B, Fedorowicz Z (2012). Antiemetic treatment for acute gastroenteritis in children: an updated Cochrane systematic review with meta-analysis and mixed treatment comparison in a Bayesian framework. BMJ Open.

[14] Weghorst AAH, Holtman GA, Wolters PI (2021). Recommendations for clinical research in children presenting to primary care out-of-hours services: a randomised controlled trial with parallel cohort study. BJGP Open.

[15] Belo JN, Bos M, Brühl F (2014). NHG — Standaard acute diarree [National Health Guideline — Standard acute diarrhea]. Huisarts Wet.

[16] Freedman SB, Ali S, Oleszczuk M (2013). Treatment of acute gastroenteritis in children: an overview of systematic reviews of interventions commonly used in developed countries. Evid Based Child Health.

[17] Tomasik E, Ziółkowska E, Kołodziej M, Szajewska H (2016). Systematic review with meta-analysis: ondansetron for vomiting in children with acute gastroenteritis. Aliment Pharmacol Ther.

[18] Geneesmiddel Ondansetron Kinderformularium [Medicine Ondansetron Children’s Formularium]. https://www.kinderformularium.nl/geneesmiddel/30/ondansetron.

[19] Kool M, Elshout G, Moll HA (2013). Duration of fever and course of symptoms in young febrile children presenting with uncomplicated illness. J Am Board Fam Med.

[20] Lisman-van Leeuwen Y, Spee LAA, Benninga MA (2013). Prognosis of abdominal pain in children in primary care — a prospective cohort study. Ann Fam Med.

[21] Hughes RA, Heron J, Sterne JAC (2019). Accounting for missing data in statistical analyses: multiple imputation is not always the answer. Int J Epidemiol.

[22] Hagbom M, Novak D, Ekström M (2017). Ondansetron treatment reduces rotavirus symptoms — a randomized double-blinded placebo-controlled trial. PLoS One.

[23] Hendrickson MA, Zaremba J, Wey AR (2018). The use of a triage-based protocol for oral rehydration in a pediatric emergency department. Pediatr Emerg Care.

[24] Freedman SB, Willan AR, Boutis K (2016). Effect of dilute apple juice and preferred fluids vs electrolyte maintenance solution on treatment failure among children with mild gastroenteritis: a randomized clinical trial. JAMA.

[25] Shanley L, Mittal V, Flores G (2013). Preventing dehydration-related hospitalizations: a mixed-methods study of parents, inpatient attendings, and primary care physicians. Hosp Pediatr.

